# A209 EXPERT CONSENSUS CRITERIA AND PRACTICAL RECOMMENDATIONS FOR PBC CARE IN THE COVID-19 ERA AND BEYOND

**DOI:** 10.1093/jcag/gwab049.208

**Published:** 2022-02-21

**Authors:** G Hirschfield, M Berenguer, A E Kremer, D Jones, V Leroy, F Adekunle, M Carbone

**Affiliations:** 1 Toronto Centre for Liver Disease, Toronto General Hospital, Toronto, ON, Canada; 2 Hepatology & Liver Transplant Unit, Le Fe University Hospital and Ciberehd, IIS La Fe, Universidad De Valencia, Valencia, Spain; 3 Friedrich Alexander University of Erlangen-Nurnberg, Erlangen, Germany; 4 Newcastle University, Newcastle upon Tyne, United Kingdom; 5 Hepatology, Henri Mondor Hospital, Creteil, France; 6 Intercept Pharmaceuticals Inc, New York, NY; 7 Division of Gastroenterology and Center for Autoimmune Liver Diseases, San Gerardo Hospital, Department of Medicine and Surgery, University of Milano-Bicocca, Monza, Italy

## Abstract

**Background:**

Primary biliary cholangitis (PBC) is a chronic autoimmune cholestatic liver disease that can progress to liver fibrosis and cirrhosis, and requires timely diagnosis, optimal treatment, and risk stratification. Several guidelines for the management of PBC have been published, including the American Association for the Study of Liver Disease (AASLD) and European Association for the Study of the Liver (EASL) Clinical Practice Guidelines, which include goals for standards of PBC care. However, recent audits have identified deficiencies in real-world PBC care. In addition, the global coronavirus (COVID-19) pandemic has generally reduced access to care, diminished healthcare resources and accelerated the use of remote patient management. There is therefore a need for simple, actionable guidance that physicians can implement in order to maintain standards of care in PBC in the new environment.

**Aims:**

A working group of ten PBC specialists from Europe and Canada were convened by Intercept Pharmaceuticals in January 2020 with the aim of defining key criteria for the care of patients with PBC.

**Methods:**

Following the outbreak of the COVID-19 pandemic, based on these criteria, a smaller working group of six PBC specialists developed practical recommendations to assist physicians in maintaining standards of care and to guide remote management of patients.

**Results:**

The working group defined five key criteria for care in PBC, encompassing PBC diagnosis, initiation of first line therapy with ursodeoxycholic acid (UDCA), risk stratification on UDCA, symptom management, and initiation of 2L therapy. The group developed 21 practical recommendations for the management of patients with PBC in the COVID-19 environment including modality, frequency and timing of investigations and monitoring. (**Figure 1**).

**Conclusions:**

The delivery of PBC care during the COVID-19 pandemic carries significant challenges. These consensus criteria and practical recommendations provide guidance for the management of PBC during the pandemic era and beyond.

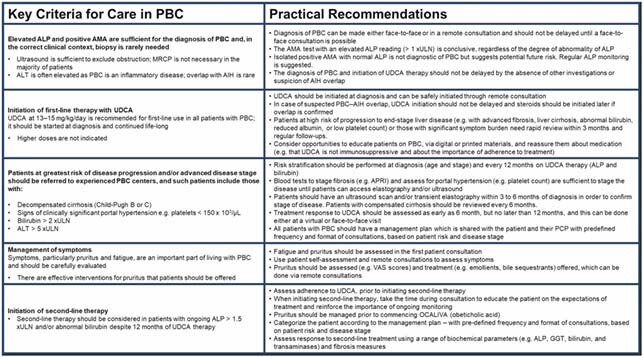

**Funding Agencies:**

NoneIntercept Pharmaceutical

